# Brain pharmacokinetics of two BBB penetrating bispecific antibodies of different size

**DOI:** 10.1186/s12987-021-00257-0

**Published:** 2021-06-02

**Authors:** Rebecca Faresjö, Gillian Bonvicini, Xiaotian T. Fang, Ximena Aguilar, Dag Sehlin, Stina Syvänen

**Affiliations:** 1 Department of Public Healt and Caring Sciences, Uppsala University, Rudbeck Laboratory, Dag Hammarskjölds väg 20, 751 85 Uppsala, Sweden; 2 BioArctic AB, Warfvinges väg 35, 112 51 Stockholm, Sweden; 3 Department of Radiology and Biomedical Imaging, Yale University, Yale PET Center, 801 Howard Avenue, New Haven, CT 06520 USA

**Keywords:** Bispecific antibody, Brain pharmacokinetics, Transferrin receptor, BBB

## Abstract

**Background:**

Transferrin receptor (TfR1) mediated enhanced brain delivery of antibodies have been studied extensively in preclinical settings. However, the brain pharmacokinetics, i.e. brain entry, distribution and elimination are still not fully understood for this class of antibodies. The overall aim of the study was to compare the brain pharmacokinetics of two BBB-penetrating bispecific antibodies of different size (210 vs 58 kDa). Specifically, we wanted to investigate if the faster systemic clearance of the smaller non-IgG antibody di-scFv3D6-8D3, in comparison with the IgG-based bispecific antibody mAb3D6-scFv8D3, was also reflected in the brain.

**Methods:**

Wild-type (C57/Bl6) mice were injected with ^125^I-iodinated ([^125^I]) mAb3D6-scFv8D3 (n = 46) or [^125^I]di-scFv3D6-8D3 (n = 32) and euthanized 2, 4, 6, 8, 10, 12, 16, or 24 h post injection. Ex vivo radioactivity in whole blood, peripheral organs and brain was measured by γ-counting. Ex vivo autoradiography and nuclear track emulsion were performed on brain sections to investigate brain and parenchymal distribution. Capillary depletion was carried out at 2, 6, and 24 h after injection of [^125^I]mAb3D6-scFv8D3 (n = 12) or [^125^I]di-scFv3D6-8D3 (n = 12), to estimate the relative levels of radiolabelled antibody in brain capillaries versus brain parenchyma. In vitro binding kinetics for [^125^I]mAb3D6-scFv8D3 or [^125^I]di-scFv3D6-8D3 to murine TfR were determined by LigandTracer.

**Results:**

[^125^I]di-scFv3D6-8D3 showed faster elimination from blood, lower brain C_max_, and T_max_, a larger parenchymal-to-capillary concentration ratio, and a net elimination from brain at an earlier time point after injection compared with the larger [^125^I]mAb3D6-scFv8D3. However, the elimination rate from brain did not differ between the antibodies. The study also indicated that [^125^I]di-scFv3D6-8D3 displayed lower avidity than [^125^I]mAb3D6-scFv8D3 towards TfR1 in vitro and potentially in vivo, at least at the BBB**.**

**Conclusion:**

A smaller size and lower TfR1 avidity are likely important for fast parenchymal delivery, while elimination of brain-associated bispecific antibodies may not be dependent on these characteristics.

**Supplementary Information:**

The online version contains supplementary material available at 10.1186/s12987-021-00257-0.

## Background

The market for biological drugs such as peptides, proteins and monoclonal antibodies (mAbs), is growing in parallel to traditional small molecule drugs. Within the field of oncology and autoimmune diseases, the use of therapeutic mAbs has advanced rapidly in the last decade [1–5]. However, biological drugs for brain diseases face the problem of very limited brain uptake due to their large size and polarity. We have shown in multiple studies using radiolabelled antibodies that less than 0.05% of the initial dose is found in the brain 2 h after i.v. or i.p. injection [6–9]. The brain access of antibodies is mainly restricted by the blood–brain barrier (BBB), a highly regulated unit of endothelial-cell tight junctions, pericytes and astrocytic end-feet [10]. Transport from the blood into the brain via the cerebrospinal fluid (CSF) is also indirectly restricted by the blood-cerebrospinal fluid barrier at the epithelium of the choroid plexus [11]. Despite the low brain penetrance of mAbs, immunotherapies are being explored for central nervous system (CNS) diseases. For neurodegenerative diseases, such as Alzheimer’s disease (AD) and Parkinson’s disease (PD), only symptomatic treatment is presently available. Antibodies have been investigated as potential disease modifying immunotherapeutic treatments for AD and PD, and some have reached advanced clinical trials [12, 13]. Due to difficulties measuring brain parenchymal concentrations of drugs, CSF is often used as a surrogate for brain interstitial fluid (ISF). However, CSF-to-serum ratios for antibodies are likely to be higher than ISF-to-plasma ratios, and hence, reported brain uptake of antibodies may be overestimated [2, 14]. Increased brain penetrance of antibody-based therapies could potentially improve both efficacy and safety of immunotherapies for CNS disorders.

During the last decade, BBB penetrating biological molecules have been developed. One of the most widely used strategies includes receptors expressed at the BBB for receptor-mediated transcytosis (RMT) of macromolecules. The insulin receptor and transferrin receptor 1 (TfR1) are the most studied RMT systems [15]. Transferrin receptor mediated transcytosis has been studied for antibodies in various formats, and has been found to be very efficient for increasing brain uptake of antibodies [1–6, 16, 17]. Antibodies are complex macromolecules, able to recognise and interact with targets in a highly specific way. They are also easily engineered into unnaturally existing formats including bispecific targeting. We have previously studied the brain distribution of different variants of mAb158, an antibody directed against soluble aggregates of amyloid-beta (Aβ), conjugated to 8D3, an anti-mouse TfR1 antibody [18–20]. This conjugation increased brain concentrations of mAb158 significantly and radiolabelling of the bispecific antibody enabled imaging of intra-brain Aβ pathology by positron emission tomography (PET) and single photon emission computed tomography (SPECT) in transgenic mice overexpressing Aβ [4, 6, 21–24].

With emerging therapies based on brain-penetrating antibodies and antibody fragments, there is an urgent need to understand the mechanisms responsible for their brain entry, intrabrain distribution, and clearance. In general, the pharmacokinetics of macromolecules in the brain is not yet well understood. It has been shown that macromolecules in the CSF can access the extracellular spaces at the brain surface by diffusion, and further, that macromolecules can be transported by convection via the perivascular spaces (PVS) of blood vessels and microvasculature to access deeper brain regions [25]. Potentially, perivascular transport also allows parenchymal access in conjunction to the microvasculature of the brain, where exchange is thought to occur from peri-arterial to peri-venous capillaries via diffusion across the parenchyma [25, 26]. Antibodies designed for brain TfR1-mediated transcytosis by fusion of a TfR1-binding moiety to the original IgG antibody enter the parenchyma primarily through the endothelial cells of the BBB. These bispecific antibodies have been shown to access the brain to a higher extent and to be more uniformly distributed inside the brain compartment and parenchyma compared with unmodified IgG antibodies [9, 21]. However, the size of the antibody construct could impact its ability to diffuse within the limited space between the cells of the parenchyma. For example, brain distribution from the CSF was shown to be size-dependent when comparing a 15 kDa single domain antibody (sdAb) and an 150 kDa IgG antibody, where the former, smaller antibody showed a more diffuse and deeper distribution into the parenchyma [25]. However, there is a general lack of knowledge regarding how size and other factors may influence the transport processes and intrabrain distribution after systemic administration of antibody drugs, including antibodies that enter the brain via TfR1 mediated BBB transcytosis.

**Fig.1:**
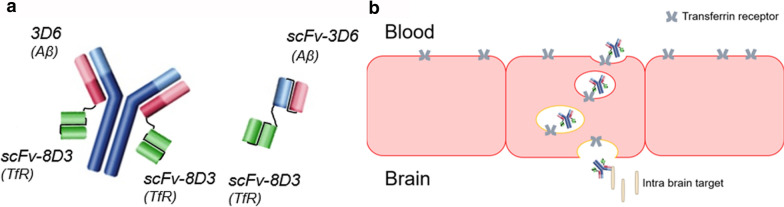
**a** Bispecific fusion antibody mAb3D6-scFv8D3 (210 kDa) and di-scFv3D6-8D3 (58 kDa). **b** Schematic illustration of transferrin-receptor mediated transcytosis at the blood–brain barrier of a bispecfic antibody

In this paper, we compared the brain pharmacokinetics of two bispecific TfR1-binding antibodies (Fig. 1) during the first 24 h after administration in wild-type mice. These bispecific antibodies were based on 3D6, the murine version of the Aβ-targeting antibody bapineuzumab, and 8D3, the TfR1 rat-anti-mouse antibody [27]. The bispecific antibodies differed fourfold in size: mAb3D6-scFv8D3, a 210 kDa IgG-like fusion antibody and di-scFv3D6-8D3, a 58 kDa fusion of two single-chain variable fragments (scFv). The smaller antibody was initially developed to be used as an antibody-based PET-ligand and is characterised by a faster clearance from blood compared with antibody constructs based on full-sized IgGs [28, 29]. The faster systemic clearance can be explained by its smaller size and lack of an Fc region, which interacts with the neonatal Fc receptor (FcRn) that prolongs antibody residence time in blood by a recycling mechanism [30]. In the present study, we asked if the faster systemic clearance of di-scFv3D6-8D3 is also reflected in the brain, and compared its brain pharmacokinetics with the brain pharmacokinetics of the larger IgG-like construct, mAb3D6-scFv8D3.

We show that the two bispecific antibodies differ in apparent brain pharmacokinetic behaviour and propose possible reasons for this.

## Methods

### Recombinant expression and purification of bispecific antibodies

The two bispecific antibodies, mAb3D6-scFv8D3 and di-scFv3D6-8D3, were cloned, expressed and purified by affinity chromatography according to previously published protocols [6, 28, 31]. Additionally, to improve the purity of di-scFv3D6-8D3, a polishing step was added by using ion exchange chromatography (IEX) on a 1 ml HiTrap SP column (GE Healthcare, Uppsala Sweden) with 50 mM HEPES as binding buffer (ionic strength 0.008 M, pH 7.0), and eluted in the same buffer with the addition of a NaCl gradient up to 0.5 M. The size and purity was determined by SDS-PAGE (see Additional file 1: Fig. S1).

### Radiochemistry

The two bispecific antibodies, mAb3D6-scFv8D3 and di-scFv3D6-8D3, were radiolabelled by direct iodination with iodine-125 (^125^I) using the Chloramine-T method [32]. In short, mAb3D6-scFv8D3, or di-scFv3D6-8D3, was mixed with ^125^I stock solution (Perkin-Elmer Inc Waltham, MA, USA) and 5 μg Chloramine-T (Sigma Aldrich, Stockholm, Sweden) in PBS to a final volume of 110 μl. The reaction was incubated for 90 s at room temperature, and then quenched with 10 μg sodium metabisulfite (Sigma Aldrich). The product was diluted to 500 μl with PBS, and separated from free-iodine and low molecular weight products with a NAP-5 size exclusion column (GE Healthcare, Uppsala, Sweden) with a total elution volume of 1 ml PBS.

### In vitro validation of radiolabelled bispecific antibodies

ELISA was performed as an in vitro quality control in order to assess the potential effect of radiolabelling on mAb3D6-scFv8D3 and di-scFv3D6-8D3. Indirect ELISA was used to measure mAb3D6-scFv8D3 and di-scFv3D6-8D3 binding to Aβ_42_ before and after ^125^I-iodination, as described previously [4]. For murine TfR1 (mTfR1) binding before and after radiolabelling, indirect ELISA was used for mAb3D6-scFv8D3 and a competition ELISA was used for di-scFv3D6-8D3 [6]. All of the ELISA setups are described in more detail in the Additional file 1: Figs. S2 and S3.

### LigandTracer-based real-time binding kinetics assay

Binding to mTfR1 was also investigated after iodine radiolabelling with LigandTracer (Ridgeview Instruments AB, Uppsala, Sweden). A small circular area in a high binding petri dish was coated with 200 nM of mTfR1 (BioArctic AB, Stockholm, Sweden) and incubated overnight at 4 °C. The plate was blocked with 5% BSA for 1 h at room temperature. The blocking buffer was replaced with 3 ml of 0.1% BSA solution and the plate was placed in a LigandTracer Grey instrument (Ridgeview Instruments AB) for a 10 min baseline measurement at room temperature. Two association phases were then measured: first with 2 nM of ^125^I-labelled antibody for 3 h and the second with 6 nM of ^125^I-labelled antibody for 4 h. The association phases were then followed by a dissociation phase overnight.

Binding kinetics were analysed with TraceDrawer software version 1.8.1 (Ridgeview Instruments AB). [^125^I]mAb3D6-scFv8D3 binding curves were analysed with a 1:1 binding model while [^125^I]di-scFv3D6-8D3 binding curves were analysed with a 1:2 binding model.

### Animals

Both male and female wild-type mice (C57/Bl6) at the age of 2.5–4 months were used for the ex vivo pharmacokinetic study (n = 78) and for capillary depletion (n = 24). The animals were housed in an approved animal facility at Uppsala University with ad libitum access to food and water. All described procedures were approved by the Uppsala Country Animal Ethics board (5.8.18–13350/2017) following the legislation and regulations of the Swedish Animal Welfare Agency and European Communities Council Directive of 22 September 2010 (20103/EU) (Table 1).

**Table 1:** The total number of animals administered [^125^I]mAb3D6-scFv8D3 or [^125^I]di-scFv3D6-8D3, respectively, with the capillary depletion animals included in brackets

Euthanization time (h) post administration	No. of individuals (n) administered [^125^I]mAb3D6-scFv8D3 (capillary depletion animals)	No. of individuals (n) administered [^125^I]di-scFv3D6-8D3 (capillary depletion animals)
2	6 (+ 4)	4 (+ 4)
4	4	4
6	6 (+ 4)	4 (+ 4)
8	7	4
10	6	4
12	6	4
16	5	4
24	6 (+ 4)	4 (+ 4)
Total	46 (+ 12)	32 (+ 12)

### Ex vivo study of ^125^I-iodinated bispecific antibodies

The molar activities attained after ^125^I-iodination of the antibodies were 90 ± 7 MBq/nmol for [^125^I]mAb3D6-scFv8D3 and 97 ± 9 MBq/nmol for [^125^I]di-scFv3D6-8D3, respectively. Mice were intravenously (i.v.) injected in the tail vein with either 0.40 ± 0.11 MBq [^125^I]mAb3D6-scFv8D3 (0.09 mg/kg) (n = 46) or 0.52 ± 0.14 MBq [^125^I]di-scFv3D6-8D3 (0.02 mg/kg) (n = 32). Blood samples (8 µL) were obtained from the tail vein of selected individuals at 1, 2, 4, 6, 8 and 10 h to investigate the concentration profile in blood. At 2, 4, 6, 8, 10, 12, 16 or 24 h after administration of radiolabelled antibody, mice were anaesthetized with isoflurane and a terminal blood sample was taken from the heart, followed by transcardial perfusion with 40 ml of 0.9% NaCl for 2.5 min to clear the brain and organs of blood. Thereafter, lung, liver, kidney, whole heart, pancreas, spleen, femoral muscle, femoral bone, skull bone and submandibular glands were isolated to investigate the biodistribution of the radiolabelled antibodies. The brain was divided into left and right hemispheres, and the left hemisphere was further divided into cerebrum and cerebellum. The brain samples were immediately frozen at − 80** °**C. The radioactivity of all samples was measured with a γ-counter (2480 Wizard™, Wallac Oy PerkinElmer, Turku, Finland). Antibody concentrations were expressed as percent of injected dose per gram tissue (%ID/g) or percent of injected dose corrected for body weight (bw) of the animal (%ID/g/bw).

1 $$\mathrm{\%}\frac{\mathrm{ID}}{\mathrm{g}}= \frac{\mathrm{measured\,radioactivity\,per\,gram\,tissue}}{\mathrm{total\,injected\,radioactivity}}.$$

2 $$\mathrm{\%}\frac{\frac{\mathrm{ID}}{\mathrm{g}}}{\mathrm{bw}}= \frac{\mathrm{measured\,radioactivity\,per\,gram\,tissue}}{\mathrm{total\,injected\,radioactivity\,per\,gram\,animal}}.$$

Brain-to-blood ratio was calculated as:

3 $${\rm Brain}{\text -}{\rm to}{\text -}{\rm blood\,ratio}= \frac{\mathrm{Brain\,radioactivity\,per\,gram\,brain}}{\mathrm{Blood\,radioactivity\,per\,gram\,blood}}.$$

The pharmacokinetic parameter estimates were based on ex vivo measured radioactivity in the cerebrum of the left hemisphere. Autoradiography, immunofluorescence and nuclear track emulsion were performed on brain sections prepared from the right hemisphere.

### Pharmacokinetic parameter estimates

Estimation of whole blood half-lives was done in Prism 8 software (GraphPad Software, Inc., La Jolla, CA, USA) using nonlinear regression with a two-phase decay model. The plateau was constrained to zero and Y0 was fixed to 50% for both antibodies, assuming an average mouse blood volume of 2 ml [33]. The fast and slow half-life _blood_ for [^125^I]mAb3D6-scFv8D3 could be estimated using 95% confidence intervals, while 90% confidence intervals were used for [^125^I]discFv3D6-8D3. The same software was used for the brain half-life estimates using nonlinear regression, and the “plateau followed by one phase decay” model to estimate the half-life during the brain net elimination phase. The plateau was constrained to zero, and the X0 was constrained to respective observed T_max_. The antibody exposure in blood and brain, AUC_blood(2–24 h)_ and AUC_brain(2–24 h)_, were quantified from the %ID/g _blood_ and %ID/g _brain_ curves, respectively, using the “area under the curve” calculations in Prism 8 (GraphPad Software).

### Within blood distribution

In addition to the BBB, TfR1 is expressed peripherally, e.g. to a high extent on immature red blood cells [34]. This can potentially affect the pharmacokinetics of bispecific antibodies. Therefore, the antibody concentration in plasma versus blood cells was investigated at the different terminal time points.

The terminal blood sample was placed in an Eppendorf tube prepared with heparin. The tube was centrifuged for 5 min at 10,000×*g* at 4 °C. Plasma was carefully separated from the blood cells by aspiration, and radioactivity in the separated fractions was measured with a γ-counter (PerkinElmer). The percentage of radioactivity in plasma was calculated by:

4 $$\mathrm{\%Plasma }= \frac{{\mathrm{Radioactivity}}_{plasma}}{{\mathrm{Radioactivity}}_{plasma} + {\mathrm{Radioactivity}}_{blood cells}}.$$

### Capillary depletion

Capillary depletion was performed on perfused mice euthanized 2, 6 or 24 h post injection of [^125^I]mAb3D6-scFv8D3 (injected dose: 0.60 ± 0.11 MBq) or [^125^I]di-scFv3D6-8D3 (injected dose: 0.56 ± 0.10 MBq). The number of animals was four per time point and antibody (total n = 24). The capillary depletion procedure was done as previously described [35, 36], with modifications as described below. Brain cortices were isolated immediately after transcardial perfusion. The cortices were weighed and homogenised in 0.8 ml cold physiological buffer (10 mM HEPES, 141 mM NaCl, 4 mM KCl, 2.8 mM CaCl_2_, 1 mM MgSO_4_, 1 mM NaH_2_PO_4_, 10 mM D-glucose adjusted to pH 7.4) with 6 strokes in an ice cold Dounce homogeniser. Thereafter, 1.6 ml of 30% Ficoll 400 (Sigma Aldrich) was added followed by an additional stroke. The homogenate was transferred to a 15 ml Falcon tube, which was centrifuged at 5200×*g* for 20 min at 4 °C. The centrifugation resulted in a parenchymal supernatant with a layer of fat, and a capillary enriched pellet which was carefully separated from the supernatant. The fractions were investigated under light microscopy with trypan blue staining to confirm enrichment of capillaries in the pellet and capillary depletion in the parenchymal supernatant. The activity of the fractions was measured in a γ-counter (PerkinElmer) and the radioactive signal for each fraction was normalised to the injected dose (%ID) in MBq.

5 $$\mathrm{\%ID }= \frac{{\mathrm{Radioactivity}}_{fraction}}{\mathrm{Injected\,radioactivity}}.$$

### Ex vivo Autoradiography

The frozen right hemispheres were sectioned sagittally (20 μm) in a cryostat (CM1850, Leica Biosystems, Nussloch, Germany) and mounted on glass slides. Duplicate sections from each animal together with a standard of ^125^I with known radioactivity were exposed to a phosphor imaging plate (MS, Multisensitive, PerkinElmer, Downers Grove, IL, USA) for 7 days. The plates were scanned in a Cyclone Plus phosphor imager (PerkinElmer) at 600 dots per inch. The generated digital image was converted with a lookup table (Royal) in ImageJ. The radioactivity standards were used to normalise intensities for images obtained from different plates (see Additional file 1: Fig. S4).

### Immunofluorescence CD31-staining

Adjacent sagittal brain cryosections to the autoradiography sections described above, were fixed in ice-cold MeOH for 10 min and then washed in PBS. The sections were blocked for 1 h with 5% Normal Goat Serum, washed in PBS, followed by a wash in 0.1% Tween-20 in PBS for 5 min. 1.25 μg/ml of the primary antibody rat-anti-mouse CD31 (BD, #553370), was applied to the sections and incubated overnight at 4 °C with slow shaking. After incubation, the sections were washed in PBS and 10 μg/ml secondary antibody goat-anti-rat (Alexa 555 or Alexa 647) was added for 1 h with slow shaking, followed by another PBS wash. The sections were stored in PBS until the nuclear track emulsion procedure (described below) was performed on the same day.

### Nuclear track emulsion

Nuclear track emulsion (NTE) allows visualisation of radiolabelled molecular compounds as silver grains on top of immuno-stained sections. NTE experiments were done in darkness, as previously described [21]. In brief, ILFORD K5 emulsion (Oxford Instruments, Gometz la Ville, France) was prepared in a 40 °C water bath as a 50:50 emulsion in MQ-water. The CD31-stained sections were immersed in the emulsion for 10 s. The sections were left to air dry for 2 h, before they were incubated in dark conditions for 5 weeks at 4 °C. The sections were developed according to the manufacturer’s instructions, then dehydrated in increasing EtOH concentration gradient (70%, 95%, 100%) and mounted with Pertex (Histolab). Images of the developed emulsion and CD31-immunofluorescent stained sections were acquired with a Zeiss Observer Z.1 microscope (Carl Zeiss Microimaging GmbH, Jena, Germany) and processed equally using the ZEN software. An alternative version of Fig. 7a, b, can be found in the supplementary material with colors converted by the function “Selective Color” in Photoshop, to show silver grains in white on a dark background (Additional file 1: Fig. S6).

### Image quantification

The percentage area of NTE-signal associated with capillary and parenchymal regions was quantified with Fiji (ImageJ) using 18–20 images per time point (1–2 individuals per time point). A standardized macro was used to process 10 sets of images from each individual at a time.

In short, rolling ball background subtraction was applied to all images. The standardized macro used pre-defined processing and thresholds to create binary images with separate signal thresholds for capillaries (CD31-image) and nuclear track emulsion (NTE-image). The total area of regions of interest (ROIs) in the NTE-image were measured using “Analyse particles”, set to count ROIs of 2–200 pixels. Next, the “Image calculator” was used to subtract the corresponding CD31-ROIs from the image. This produced an image with NTE signal only in the non-capillary regions (parenchymal). NTE-image ROIs were measured again with “Analyse particles”, yielding a total area of NTE-signal in the parenchymal regions. The percentage area of NTE-signal in the parenchymal regions was calculated by dividing the total area after subtraction with the total area before subtraction × 100. Capillary signal was calculated by 1 – parenchymal signal. The accuracy of the ROIs was evaluated visually by using overlays on the original image, as shown in Additional file 1: Fig. S5.

### Statistical analysis

Data is presented as mean ± standard deviation, if not stated otherwise. All calculations were made in Prism 8 (GraphPad Software, Inc.). The EC50 values for the ELISAs were calculated from agonist vs response curves, while the IC50 was calculated from the inhibitor vs response curves. A paired t-test was used to compare differences within the EC50 values or the IC50 values. The correlation between mean intensity of the ex vivo autoradiography and the injected dose per gram brain was evaluated with Pearson’s correlation coefficient. Two-way analysis of variance followed by Bonferroni’s post hoc test was used to test differences in plasma or capillary distribution between [^125^I]mAb3D6-scFv8D3 and [^125^I]di-scFv3D6-8D3 at the different time points.

## Results

### Size and purity assessed by SDS-PAGE

The size and purity of mAb3D6-scFv8D3 and di-scFv3D6-8D3 was analysed by SDS-PAGE under non-reducing conditions. The SDS-PAGE gels showed bands at the expected size around 210 kDa for mAb3D6-scFv8D3 and 58 kDa for di-scFv3D6-8D3. The di-scFv3D6-8D3 preparation contained less impurities in the higher molecular weight range after HiTrap-IEX purification (Additional file 1: Fig. S1).

### In vitro affinity after radiolabelling

The antibodies retained binding to the antigens after radiolabelling. The Aβ ELISA showed no difference between radiolabelled and non-radiolabelled antibody. The mTfR1 ELISA indicated that the affinity of the radiolabelled antibody was on average twofold lower than the non-radiolabelled version. However, none of the differences were statistically significant (n = 3 repetitions for all assays) (Additional file 1: Figs S2 and S3).

**Fig.2:**
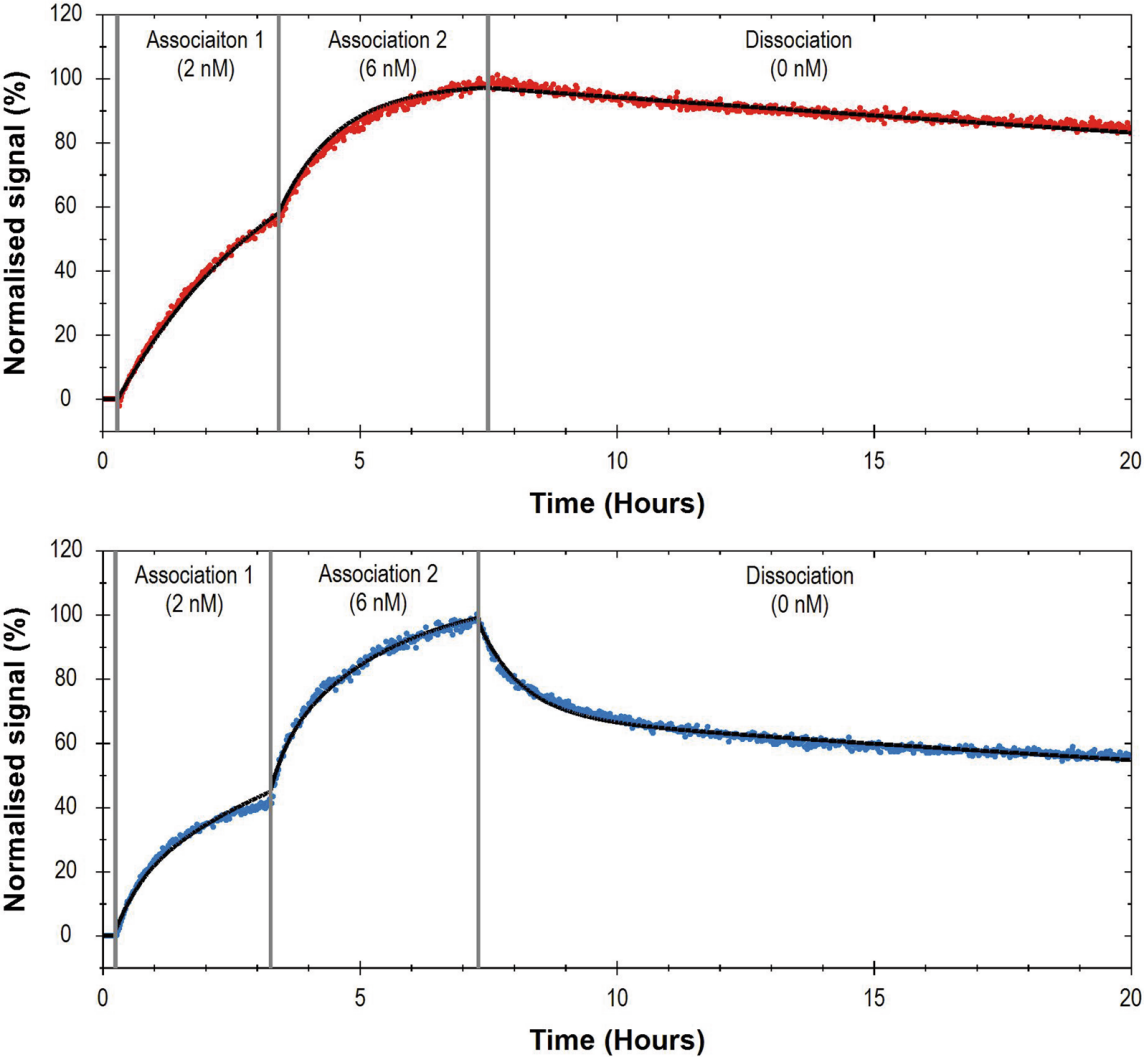
LigandTracer-based real-time kinetics of [^125^I]mAb3D6-scFv8D3 (red) and [^125^I]di-scFv3D6-8D3 (blue) binding to mTfR1. Recording included 10 min of baseline, 3 h of association phase 1 with 2 nM of ^125^I-labeled antibody, 4 h of association phase 2 with 6 nM of ^125^I-labeled antibody, and a dissociation phase overnight. The bold line represents the fitted curve from a 1:1 binding model for [^125^I]mAb3D6-scFv8D3 and a 1:2 binding model for [^125^I]di-scFv3D6-8D3

The LigandTracer indicated a fivefold stronger binding of [^125^I]mAb3D6-scFv8D3 to mTfR1 compared with [^125^I]di-scFv3D6-8D3 based on the K_D_1 estimates (Table 2). A 1:1 model was fitted to the [^125^I]mAb3D6-scFv8D3 LigandTracer data, whereas a 1:2 model proved to be the best fit for the [^125^I]di-scFv3D6-8D3 data (Fig. 2).

**Table 2:** LigandTracer binding kinetic parameters of fitted data for [^125^I]mAb3D6-scFv8D3 and [^125^I]di-scFv3D6-8D3

Ligand	Model	Bmax1 (norm %)	k_a1_ (1/Ms)	k_d1_ (1/s)	K_D1_ (M)
[^125^I]mAb3D6-scFv8D3	1:1	98.73 ± 2.37	3.73 × 10^4^ ± 7.75 × 10^3^	1.91 × 10^–6^ ± 1.01 × 10^–6^	5.38 × 10^–11^ ± 2.92 × 10^–11^
[^125^I]di-scFv3D6-8D3	1:2	76.00 ± 3.66	2.02 × 10^4^ ± 1.51 × 10^3^	5.32 × 10^–6^ ± 3.96 × 10^–7^	2.65 × 10^–10^ ± 1.39 × 10^–11^

		Bmax2 (norm %)	k_a2_ (1/Ms)	k_d2_ (1/s)	K_D2_ (M)
[^125^I]di-scFv3D6-8D3	1:2	73.14 ± 26.87	5.74 × 10^4^ ± 2.92 × 10^4^	3.61 × 10^–4^ ± 2.98 × 10^–5^	7.55 × 10^–9^ ± 3.78 × 10^–9^

Association rate constant, k_a_; dissociation rate constant, k_d_; equilibrium dissociation constant, K_D_. Values are reported as mean ± standard deviation (n = 3)

### Pharmacokinetics in blood

**Fig. 3:**
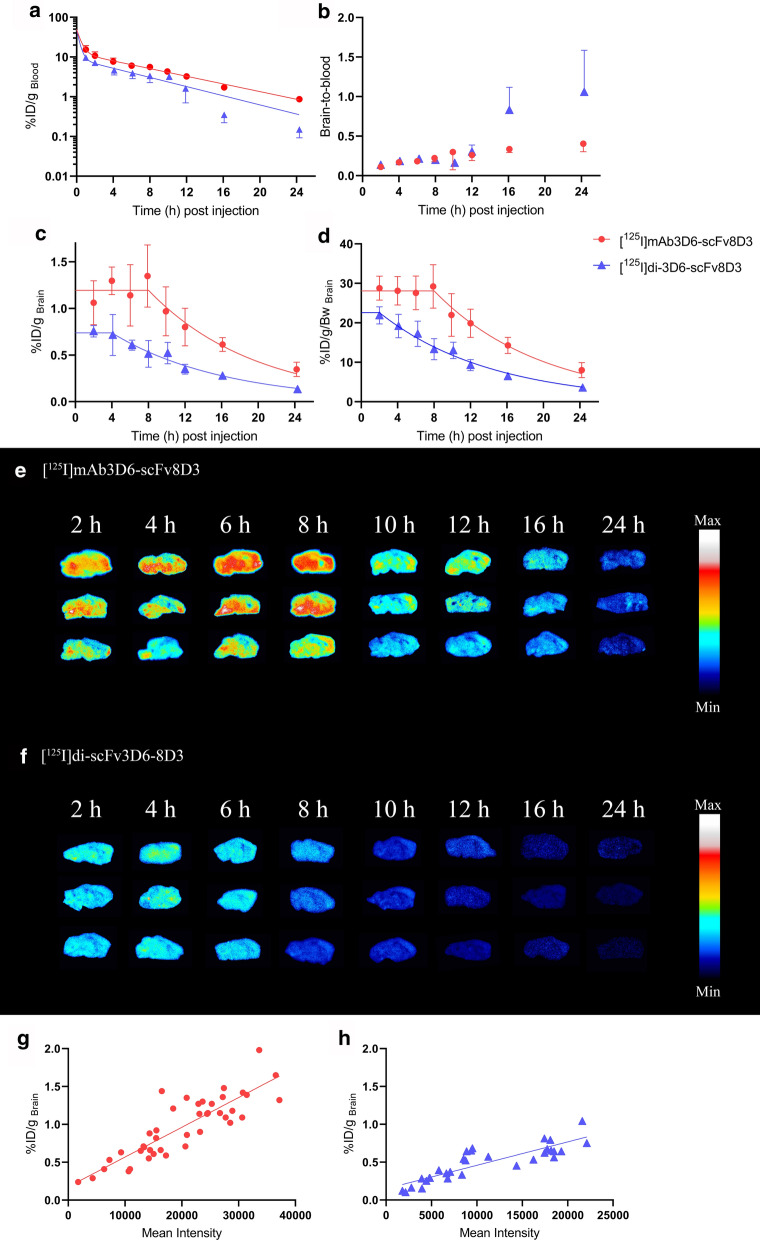
**a** Whole blood pharmacokinetics (%ID/g _blood_) of [^125^I]mAb3D6-scFv8D3 (red) and [^125^I]di-scFv3D6-8D3 (blue) and a two phase decay model fitted to the data **b** Average brain-to-blood ratio for [^125^I]mAb3D6-scFv8D3 and [^125^I]di-scFv3D6-8D3 injected animals between 2 and 24 h **c** Brain pharmacokinetics of [^125^I]mAb3D6-scFv8D3 and [^125^I]di-scFv3D6-8D3 expressed as: %Injected dose per gram brain (%ID/g _brain_). The line represents the plateau followed by one phase decay model used to estimate the brain half-lives of [^125^I]mAb3D6-scFv8D3 and [^125^I]di-scFv3D6-8D3 **d** Brain pharmacokinetics of [^125^I]mAb3D6-scFv8D3 and [^125^I]di-scFv3D6-8D3 expressed as %ID/g _brain_ normalised to body weight (bw) (%ID/g/bw), the line represents a plateau followed by one phase decay model. Values in **a–d** are reported as mean ± standard deviation. **e–f** Sagittal brain sections of three representative individuals per time point, euthanized 2, 4, 6, 8, 10, 12, 16 or 24 h after administration of [^125^I]mAb3D6-scFv8D3 or [^125^I]di-scFv3D6-8D3 **g** Correlation between mean intensity of ROIs in the autoradiography and the whole brain radioactivity concentration (%ID/g _brain_ from **c**) for all individuals administered with [^125^I]mAb3D6-scFv8D3 (red, R^2^ = 0.74, Pearsons r = 0.86 ****p < 0.0001) or [^125^I]di-scFv3D6-8D3 (blue, R^2^ = 0.74, Pearsons r = 0.86 **** p < 0.0001)

Analysis of whole blood samples showed that [^125^I]di-scFv3D6-8D3 displayed faster elimination from whole blood than [^125^I]mAb3D6-scFv8D3, both in the fast and in the slow phase (Fig. 3a; Table 3). The total whole blood exposure, AUC_blood(2–24 h)_, was 2-fold higher for [^125^I]mAb3D6-scFv8D3 compared with [^125^I]di-scFv3D6-8D3 (Table 3).

**Table 3:** Whole blood half-lives for the fast and slow phase and AUC_blood(2–24 h)_ for [^125^I]mAb3D6-scFv8D3 and [^125^I]di-scFv3D6-8D3

Ligand	Half-life (fast)	Half-life (slow)	AUC_blood(2–24 h)_
[^125^I]mAb3D6-scFv8D3	0.3 h (0.3–0.4)	6.1 h (5.7–6.6)	95.2 (85.4–104.9)
[^125^I]di-scFv3D6-8D3	0.2 h (0.2–0.3)^a^	5.2 h (4.5–6.1)^a^	53.7 (46.2–61.1)

The confidence intervals shown in brackets (CI_95%_ and ^a^CI_90%_)

### Pharmacokinetics in brain

The mean brain concentrations for [^125^I]mAb3D6-scFv8D3 and [^125^I]di-scFv3D6-8D3b at 2 h post injection were 1.1 ± 0.23%ID/g_brain_ and 0.76 ± 0.06%ID/g_brain_, respectively. Both antibodies had increased brain uptake in comparison to unmodified IgGs which have a reported brain concentration of ~ 0.03%ID/g _brain_ using a similar experimental set-up [6, 9]. The brain exposure over time, quantified as AUC_brain(2–24 h)_, was 2-fold higher for [^125^I]mAb3D6-scFv8D3 compared with [^125^I]di-scFv3D6-8D3 (Table 4). Brain-to-blood ratio for [^125^I]di-scFv3D6-8D3 diverged from [^125^I]mAb3D6-scFv8D3 after 12 h post administration and remained higher than the brain-to-blood ratio of the larger bispecific antibody for the following 12 h (Fig. 3b). Interestingly, [^125^I]mAb3D6-scFv8D3 displayed high and stable brain concentrations during the first 8 h post injection, with T_max(brain)_ occurring around 8 h, followed by a net elimination phase (Fig. 3c, d). The smaller antibody, [^125^I]di-scFv3D6-8D3, displayed an earlier T_max(brain)_ around 4 h post administration, but when corrected for body weight, the T_max(brain)_ of [^125^I]di-scFv3D6-8D3 appeared to occur already at 2 h. Overall, [^125^I]di-scFv3D6-8D3 elimination from brain began earlier than [^125^I]mAb3D6-scFv8D3 elimination. However, once brain concentrations started to decrease, the brain half-life was similar for both [^125^I]mAb3D6-scFv8D3 and [^125^I]di-scFv3D6-8D3 (Fig. 3c, d; Table 4).

**Table 4:** Estimated brain half-lives, AUC_brain(2–24 h)_ and observed brain T_max,_ of %ID/g _brain_ for [^125^I]mAb3D6-scFv8D3 and [^125^I]di-scFv3D6-8D3

Ligand	Half-life_brain_ (h)	AUC_brain(2–24 h)_	T_max_ (h)
[^125^I]mAb3D6-scFv8D3	8.1 (5.7–11.9)	18.1 (16.0–20.1)	8
[^125^I]di-scFv3D6-8D3	8.5 (6.8–11.0)	8.8 (7.9–9.7)	4

The confidence intervals are shown in brackets (CI_95%_)

The brain radioactivity concentration–time profiles were visually and qualitatively confirmed by autoradiography on sagittal brain sections from animals injected with [^125^I]mAb3D6-scFv8D3 or [^125^I]di-scFv3D6-8D3 (Fig. 3e, f). Animals administered with [^125^I]mAb3D6-scFv8D3 displayed higher radioactivity in brain sections (higher mean intensity of pixels) at later time points, while the smaller bispecific antibody, [^125^I]di-scFv3D6-8D3, showed lower brain radioactivity, in accordance with the brain concentration–time profiles (Fig. 3d). Correlation between the autoradiography mean intensities and the measured brain radioactivity concentration (%ID/g_brain_) for each individual is shown in Fig. 3g, h.

### Peripheral biodistribution

**Fig. 4:**
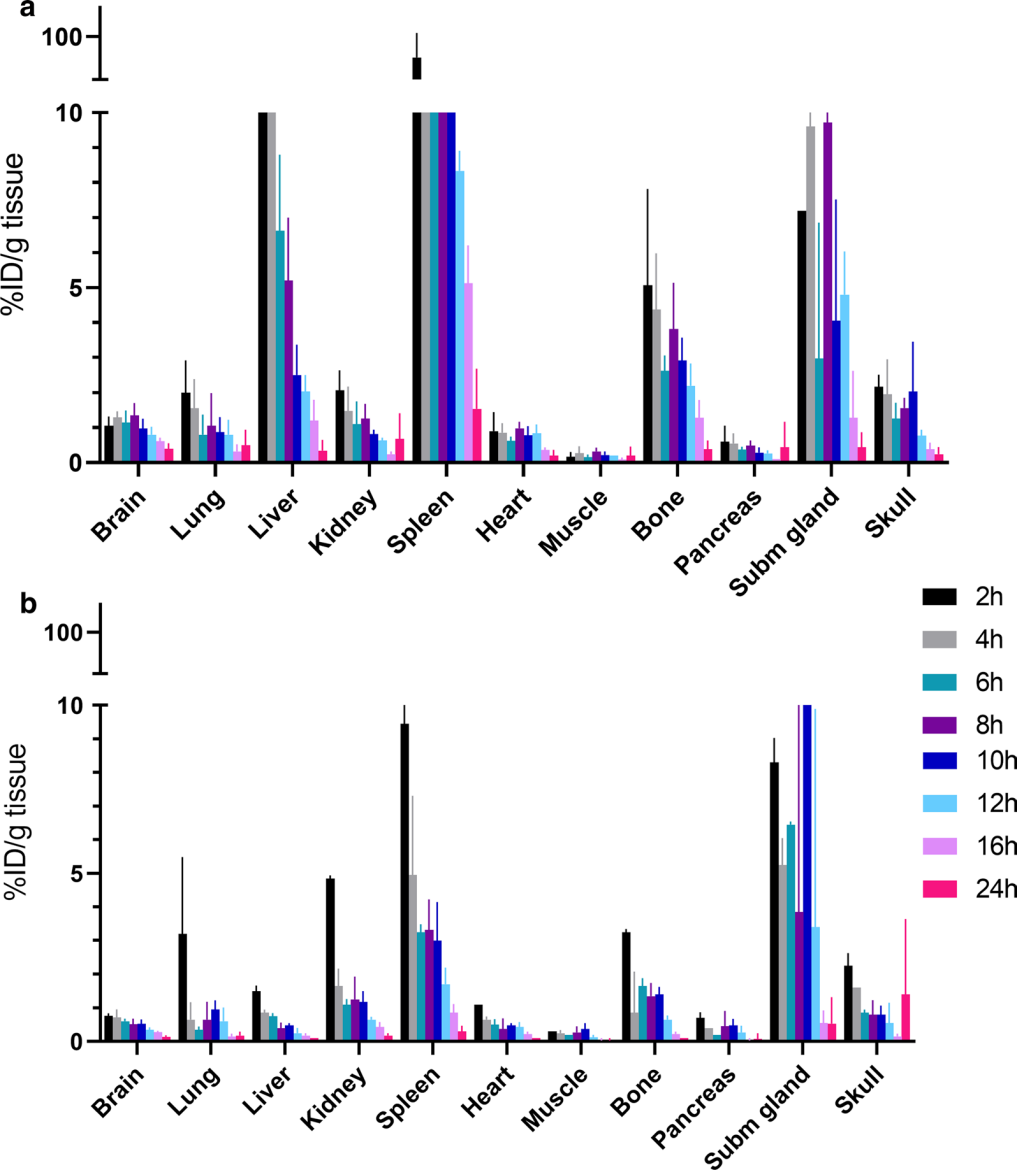
Biodistribution of **a** [^125^I]mAb3D6-scFv8D3 and **b** [^125^I]di-scFv3D6-8D3-injected animals 2–24 h post injection. Data for brain is duplicated from Fig. 3c

Distribution of the two ligands to peripheral organs was higher overall for [^125^I]mAb3D6-scFv8D3 compared with [^125^I]di-scFv3D6-8D3. Especially liver and spleen displayed higher uptake of [^125^I]mAb3D6-scFv8D3 compared with [^125^I]di-scFv3D6-8D3 (Fig. 4).

**Fig. 5:**
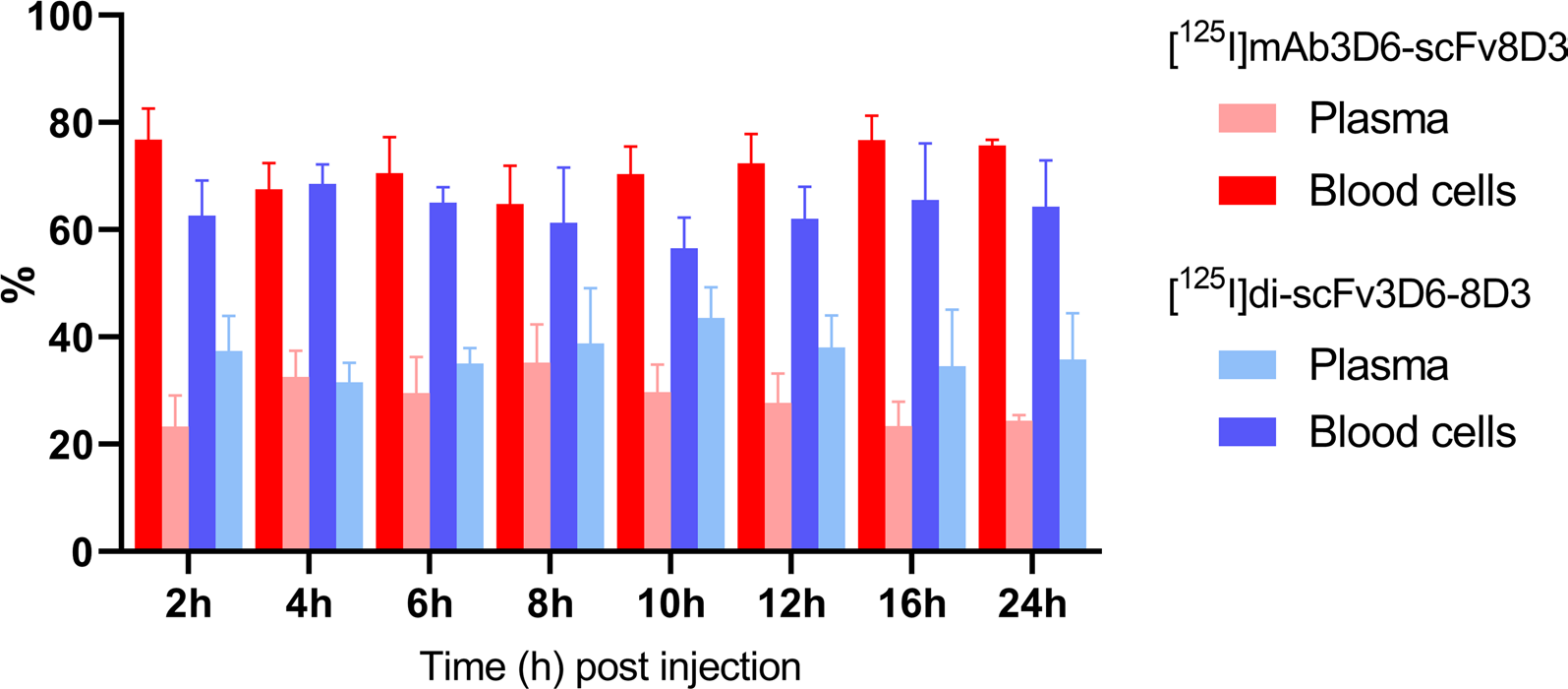
Percent of radioactivity in plasma vs blood cells 2–24 h post injection of **a** [^125^I]mAb3D6-scFv8D3 and **b** [^125^I]di-scFv3D6-8D3

The majority of the antibody molecules in whole blood were associated with blood cells rather than with plasma. On average, 72 ± 7% of [^125^I]mAb3D6-scFv8D3 and 63 ± 7% of [^125^I]di-scFv3D6-8D3 was found in the blood cell fraction over the course of the experiment. Thus, [^125^I]di-scFv3D6-8D3 showed higher relative concentrations in plasma compared with [^125^I]mAb3D6-scFv8D3, although the differences were not significant (Fig. 5).

### Parenchymal versus capillary distribution

**Fig. 6:**
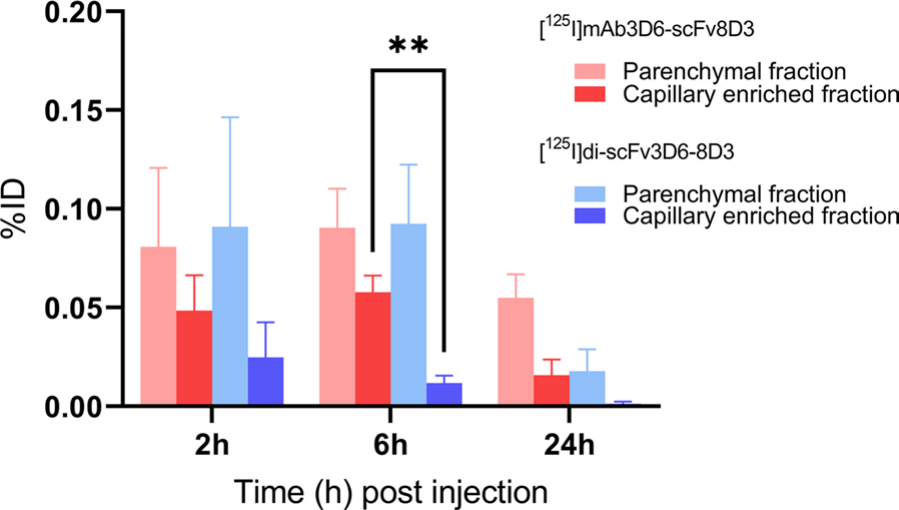
Capillary depletion for animals injected with [^125^I]mAb3D6-scFv8D3 (red) or [^125^I]di-scFv3D6-8D3 (blue). Values are expressed as percent of injected dose (%ID) for respective fraction and reported as mean ± standard deviation. The number of animals was 4 for each time point and antibody. A two-way analysis of variance followed by Bonferroni’s post hoc test was performed to compare %ID of capillary enriched fractions (**p < 0.01)

Capillary depletion of brain homogenates showed that the distribution between the capillary enriched fraction and parenchymal fraction differed between the antibodies over time (Fig. 6). [^125^I]mAb3D6-scFv8D3 was found to a higher degree in the capillary enriched fraction than [^125^I]di-scFv3D6-8D3 for all time points and was significantly higher at 6 h (p < 0.01). The amount of [^125^I]mAb3D6-scFv8D3 in the capillary enriched fraction remained high within the first 6 h post injection, but had decreased at 24 h. On the contrary, the [^125^I]di-scFv3D6-8D3 capillary enriched fraction continually decreased over time (Table 5).

**Table 5:** Distribution between parenchymal and capillary enriched fraction (%)

	[^125^I]mAb3D6-scFv8D3		[^125^I]di-scFv3D6-8D3	
	Parenchyma	Capillary	Parenchyma	Capillary
2 h	63 ± 12%	37 ± 12%	79 ± 4%	21 ± 4%
6 h	63 ± 5%	37 ± 5%	88 ± 3%	12 ± 3%
24 h	79 ± 9%	21 ± 9%	94 ± 2%	6 ± 2%

Values are shown as mean ± standard deviation

**Fig. 7:**
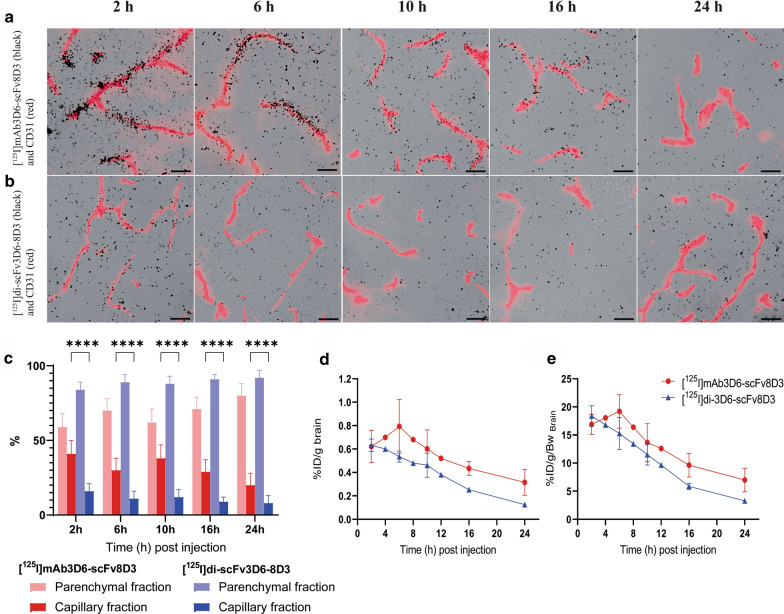
Representative images of NTE (black puncta) detecting *i.v.* injected **a** [^125^I]mAb3D6-scFv8D3 or **b** [^125^I]di-scFv3D6-8D3 and CD31-flourescent staining (red) in mouse brains. Black scale bar = 20 μm. **c** Quantification of NTE images for [^125^I]mAb3D6-scFv8D3 and [^125^I]di-scFv3D6-8D3-injected animals, the association of black puncta (antibody-derived signal) with parenchyma or capillaries in %area of black puncta. There was a significantly higher distribution to the capillaries of [^125^I]mAb3D6-scFv8D3 compared to [^125^I]di-scFv3D6-8D3 at all investigated time points, as determined by two way analysis of variance followed by Bonferroni’s post hoc test (****p < 0.0001). Values are reported as mean ± standard deviation, with 18–20 images for each time point and antibody. Brain pharmacokinetics corrected for parenchymal partitioning expressed as **d** %ID/g _brain_ and **e** %ID/g _brain_ corrected for body weight (bw) for [^125^I]mAb3D6-scFv8D3 and [^125^I]di-scFv3D6-8D3-injected animals. Values are reported as mean ± standard deviation

CD31 staining combined with NTE revealed a higher association of [^125^I]mAb3D6-scFv8D3 compared with [^125^I]di-scFv3D6-8D3 to brain capillaries throughout the 24 h study period (Fig. 7a, b).This difference was already clear 2 h post injection. Quantification of images at the 2 h time point indicated that [^125^I]mAb3D6-scFv8D3-derived signal was nearly equally distributed between parenchyma and capillaries (59 ± 9% vs 41 ± 9% respectively, Fig. 7c). In contrast, the majority of [^125^I]di-scFv3D6-8D3-derived signal 2 h post injection was found in the parenchyma (84 ± 5%, Fig. 7c). The higher distribution to the capillaries for [^125^I]mAb3D6-scFv8D3 compared to [^125^I]di-scFv3D6-8D3 was significant at all investigated time points (p < 0.0001). When brain concentration curves (Fig. 3c, d) were corrected for parenchymal/capillary distribution, [^125^I]mAb3D6-scFv8D3 and [^125^I]di-scFv3D6-8D3 displayed similar parenchymal uptake (%ID/g _brain_) 2 h post injection (Fig. 7d). After additional correction for body weight, [^125^I]di-scFv3D6-8D3 displayed slightly higher brain uptake (%ID/g/bw) compared with [^125^I]mAb3D6-scFv8D3 (Fig. 7e). The elimination from brain parenchyma for [^125^I]mAb3D6-scFv8D3 was slightly slower compared with [^125^I]di-scFv3D6-8D3 after correction for capillary-parenchyma distribution (Fig. 7d, e).

## Discussion

In this study, we compared the brain pharmacokinetics of two radiolabelled bispecific antibodies, the IgG-like [^125^I]mAb3D6-scFv8D3 fusion antibody and the non-IgG-like tandem construct of two antibody-single-chain variable fragments, [^125^I]di-scFv3D6-8D3.

The smaller [^125^I]di-scFv3D6-8D3 displayed faster elimination from blood when compared with [^125^I]mAb3D6-scFv8D3, confirming that a smaller bispecific antibody construct, lacking an Fc domain, is associated with faster pharmacokinetic processes [28]. The level of exposure was 2-fold lower both in blood (AUC_blood(2–24 h)_) and in brain (AUC_brain(2–24 h)_) for [^125^I]di-scFv3D6-8D3, thus the faster blood clearance of [^125^I]di-scFv3D6-8D3 was reflected in the brain by a proportionally lower brain exposure. In addition, peak antibody concentration in brain was observed earlier for [^125^I]di-scFv3D6-8D3 compared with [^125^I]mAb3D6-scFv8D3. Interestingly once the brain net elimination phase was initiated, the rate of elimination from brain was similar for both antibodies.

One reason for the delayed elimination phase from brain for [^125^I]mAb3D6-scFv8D3, in comparison with [^125^I]di-scFv3D6-8D3, is likely the higher degree and prolonged association with brain capillaries throughout the study period for [^125^I]mAb3D6-scFv8D3. Although [^125^I]mAb3D6-scFv8D3 showed higher total brain concentrations than [^125^I]di-scFv3D6-8D3 in general, a larger fraction of [^125^I]mAb3D6-scFv8D3 was retained in the capillaries as shown with both capillary depletion and nuclear track emulsion. These two radically different methods led to very similar results regarding the relative antibody distribution between capillaries and parenchyma. Although the total concentrations of [^125^I]di-scFv3D6-8D3 in the brain was overall lower, the smaller antibody appeared to a higher relative degree in the parenchyma. This could be due to a more efficient transcytosis across the BBB compared with [^125^I]mAb3D6-scFv8D3 and in fact, when corrected for the capillary-parenchyma partitioning, the two antibodies reached similar parenchymal concentrations 2 h post injection. The high association of [^125^I]mAb3D6-scFv8D3 to capillaries could be a result of the higher blood exposure of [^125^I]mAb3D6-scFv8D3, yielding to accumulation over time at the capillaries. However, previous studies have showed that higher affinity to mTfR1 has been linked to poorer transcytosis [17, 37]. Perhaps more importantly in this study, binding mode and the avidity, i.e. the product of the affinity of all interactions between the antibody and the target, could explain the differences observed in capillary association. The larger antibody is by design bivalent, as it contains two scFv8D3 fragments, but previous in vitro studies have shown that the large antibody format is sterically hindered from bivalent interactions with TfR [6]. In contrast, in vitro real time kinetic binding curves obtained from the LigandTracer in the present study indicated a fivefold higher K_D_ of [^125^I]mAb3D6-scFv8D3 compared with [^125^I]di-scFv3D6-8D3 towards mTfR1. This discrepancy is perhaps due to assessment of the TfR-antibody interaction at higher concentrations in this study compared to previously. We speculate that, at high TfR1 concentrations, as in the LigandTracer analyses, a portion of [^125^I]mAb3D6-scFv8D3 could simultaneously interact with two TfR molecules. Potentially, such interactions occur to some extent also in vivo, and could contribute to the observed differences in pharmacokinetic behaviour between [^125^I]mAb3D6-scFv8D3 and [^125^I]di-scFv3D6-8D3 at the BBB. Bivalent interactions have been reported to trigger clustering of the TfR-antibody complex and lysosome sorting within the endothelial cells [3, 38]. Interestingly, the antibodies displayed only a minor difference in distribution between blood cells and plasma. We speculate that TfR1 expression could be different on blood and endothelial cells, and thus, avidity could contribute to a stronger binding at the BBB but not at TfR1 expressing blood cells.

Both antibodies displayed initial brain concentrations around 1% ID/g brain which is around 30-fold higher than what has been reported for radiolabelled unmodified IgGs [6–9, 39]. Both antibodies also bound to TfR1 and human Aβ. However, since only wild-type mice were used in the present study, the impact of in vivo interaction with Aβ, for example in capillaries, is absent. Therefore, the brain pharmacokinetics studied here are likely to be relevant for other TfR1 binding antibodies, including bispecific antibodies directed towards other intra-brain targets. The rationale for using Aβ-binding bispecific antibodies was that a number of different Aβ-binding formats have been studied during the last 10 years, thus being the most frequently used application for brain penetrating bispecific antibodies [2, 3, 6, 23, 24, 40].

Despite different C_max_ and T_max_, the antibodies in this study showed similar rates of clearance during the elimination phase. This suggests that the elimination route could be the same for both antibodies. One obvious option would be reverse TfR transcytosis as suggested for transferrin, the endogenous ligand of the receptor [41]. However, since the antibody concentration is very low in the brain it is unlikely that this would be a very efficient route of elimination. In addition, it is likely that there must exist mechanisms for antibody removal from the brain that are not dependent on TfR. Some studies propose the role of reverse transcytosis by the FcRn receptor for Fc-receptor binding antibodies [42], for example for the clearance of intracranially administered IgGs in rats [43]. If this efflux at the endothelium also applies to transcytosed bispecific IgGs, it could explain why there appeared to be a prolonged retention in the capillaries of [^125^I]mAb3D6-scFv8D3. However, since [^125^I]di-scFv3D6-8D3 lacks an Fc-region, this route fails to explain how this antibody was eliminated from the brain. Perhaps the most likely route suggested for clearance of antibodies is via bulk flow to the lymphatic system [10, 44]. In 1981, Cserr and colleagues found that radiolabelled macromolecules injected in the rat brain were eliminated according to one phase elimination, with similar half-lives, despite large differences in molecular weight [45]. This supported convective bulk flow as the clearance pathway of large molecules from the brain, and the evidence for bulk flow elimination of large molecules has been discussed by others [10, 26, 44, 46]. This pathway of elimination for large molecules is consistent with the results from the present study. Radiolabelled albumin (69 kDa) injected in rat brain had a half-life in brain of 12.2 h, which is within a similar range as in the present study of two bispecific antibodies [45]. Antibodies in the parenchyma are likely transported via diffusion, but when reaching perivascular spaces the transport is mainly by bulk flow along the venous vasculature of the brain for clearance e.g. via the venous sinuses and deep cervical lymph nodes [26, 47]. A smaller format would likely be removed faster than a larger format by diffusion in the brain parenchyma, but at similar rate once reaching the bulk flow of the PVS [25, 26]. Additionally, the higher avidity of [^125^I]mAb3D6-scFv8D3 to TfR could cause prolonged association to and internalisation in parenchymal cells expressing TfR, such as neurons [2]. This could also have contributed to the slower brain elimination from parenchyma of the IgG-like antibody, after correction for capillary-parenchymal partitioning.

Pharmacokinetic studies based on radioligands benefit from the high sensitivity of radioactivity measurements, and thus, the minute amounts that can be detected and the very small sample volumes needed. However, if [^125^I]di-scFv3D6-8D3 was degraded faster than [^125^I]mAb3D6-scFv8D3 after internalisation into parenchymal cells, it could contribute to a higher parenchyma-to-capillary concentration ratio as free iodine will be eliminated to the intracellular space [48, 49]. On the other hand, deiodination and degradation would also be interpreted as removal of the antibody, and as discussed previously, elimination rate was similar for both antibodies. We have previously shown that iodine-125 radiolabelling is stable with very low levels of free iodine-125 in brain samples after antibody injection [8]. Still, it should be kept in mind that antibody and iodine-125 to some extent could be separate entities.

## Conclusions

In summary, the smaller [^125^I]di-scFv3D6-8D3 showed faster elimination from blood, a lower brain C_max_, a lower brain T_max_, a larger parenchyma-to-capillary concentration ratio, and a net elimination from brain at an earlier time point after injection compared with the larger [^125^I]mAb3D6-scFv8D3. However, the elimination rate from brain did not differ between the antibodies. The study also indicated that [^125^I]di-scFv3D6-8D3 displayed lower avidity than [^125^I]mAb3D6-scFv8D3 towards TfR1 in vitro and potentially in vivo, at least at the BBB. Thus, a small size and lower TfR1 avidity seem to be important for fast and efficient parenchymal delivery, while elimination of brain associated bispecific antibodies may not be dependent on these characteristics.

## Supplementary Information

**Additional file 1**: **Fig. S1** a Non-reducing SDS-PAGE (4-12% Bis-Tris gel) of purified di-scFv3D6-8D3 and mAb3D6-scFv8D3. Lane 1 is protein ladder (Chameleon^®^ Duo Pre-stained Protein ladder): lanes 2-4 is di-scFv3D6-8D3 purified by a HisTrap column; lanes 5-7 is di-scFv3D6-8D3 after HiTrap ion exchange chromatography; and lanes 8-10 is mAb3D6-scFv8D3 after purification by a Protein G column. For all proteins, 2, 1 and 0.5 μg was added in lanes 2-4, 5-7 and 8-10, respectively b Quantification of %Purity of lane 2 and 5, HMW = High molecular weight (>58 kDa) and LMW = low molecular weight (< 58 kDa). **Fig. S2** a Aβ_42_ (50 nM) indirect ELISA of mAb3D6-scFv8D3 and [^125^I]mAb3D6-scFv8D3 b EC50 (mean±SD) and paired t-test of n=3 repetitions Aβ (50 nM) indirect ELISA of mAb3D6-scFv8D3 and [125I]mAb3D6-scFv8D3 c mTfR1 (13.3 nM) indirect ELISA of mAb3D6-scFv8D3 and [^125^I]mAb3D6-scFv8D3 c EC50 (mean±SD) and paired t-test of n=3 repetitions mTfR1 (13.3 nM) indirect ELISA of mAb3D6-scFv8D3 and [^125^I]mAb3D6-scFv8D3. **Fig. S3** a (50 nM) indirect ELISA of di-scFv3D6-8D3 and [^125^I]di-scFv3D6-8D3 b EC50 (mean±SD) and paired t-test of n=3 repetitions Aβ (50 nM) indirect ELISA for di-scFv3D6-8D3 and [^125^I]di-scFv3D6-8D3 c mTfR1 competition ELISA of di-scFv3D6-8D3 and [^125^I]di-scFv3D6-8D3 d IC50 (mean ± SD) and paired t-test of n=3 repetitions mTfR1 competition ELISAs for di-scFv3D6-8D3 and [^125^I]di-scFv3D6-8D3. **Fig. S4** ^125^I Standards mean intensity ± SD of the plates used in the brain autoradiography experiments (1000 Bq n=7; 333 Bq n=7; 111 Bq n = 11). **Fig. S5** Example overlay image. The accuracy of the thresholding in the NTE-image quantification was evaluated visually by applying the respective ROI outlines (NTE and CD31) as overlays with 50% opacity on the original composite image. **Fig. S6** Color-inverted version of Fig. 7a-b, showing NTE (white puncta) detecting i.v. injected a [125I]mAb3D6-scFv8D3 or b [^125^I]di-scFv3D6-8D3 and CD31-flourescent staining (red) in mouse brain sections.

## Data Availability

The datasets used and/or analysed during the current study are available from the corresponding author on reasonable request.

## Abbreviations

mAb: Monoclonal antibody
CSF: Cerebrospinal fluid
BBB: Blood–brain barrier
AD: Alzheimer’s disease
PD: Parkinson’s’ disease
ISF: Interstitial fluid
RMT: Receptor mediated transcytosis
mTfR1: Mouse transferrin receptor 1
PVS: Perivascular space
sdAb: Single domain antibody
IgG: Immunoglobulin G
FcRn: Neonatal Fc-receptor
scFv: Single chain variable fragment
IEX: Ion exchange chromatography
NTE: Nuclear track emulsion
Aβ: Amyloid beta
PET: Positron emission tomography
