# Supplementary material for: Brain pharmacokinetics of two BBB penetrating bispecific antibodies of different size
Source: Fluids Barriers CNS. 2021 Jun 2;18:26. doi: 10.1186/s12987-021-00257-0 (PMC8170802; doi:10.1186/s12987-021-00257-0)
Supplement: Supplementary file 1 — Additional file 1: Fig. S1 a Non-reducing SDS-PAGE (4-12% Bis-Tris gel) of purified di-scFv3D6-8D3 and mAb3D6-scFv8D3. Lane 1 is protein ladder (Chameleon® Duo Pre-stained Protein ladder): lanes 2-4 is di-scFv3D6-8D3 purified by a HisTrap column; lanes 5-7 is di-scFv3D6-8D3 after HiTrap ion exchange chromatography; and lanes 8-10 is mAb3D6-scFv8D3 after purification by a Protein G column. For all proteins, 2, 1 and 0.5 μg was added in lanes 2-4, 5-7 and 8-10, respectively b Quantification of %Purity of lane 2 and 5, HMW = High molecular weight (>58 kDa) and LMW = low molecular weight (< 58 kDa). Fig. S2 a Aβ42 (50 nM) indirect ELISA of mAb3D6-scFv8D3 and [125I]mAb3D6-scFv8D3 b EC50 (mean±SD) and paired t-test of n=3 repetitions Aβ (50 nM) indirect ELISA of mAb3D6-scFv8D3 and [125I]mAb3D6-scFv8D3 c mTfR1 (13.3 nM) indirect ELISA of mAb3D6-scFv8D3 and [125I]mAb3D6-scFv8D3 c EC50 (mean±SD) and paired t-test of n=3 repetitions mTfR1 (13.3 nM) indirect ELISA of mAb3D6-scFv8D3 and [125I]mAb3D6-scFv8D3. Fig. S3 a (50 nM) indirect ELISA of di-scFv3D6-8D3 and [125I]di-scFv3D6-8D3 b EC50 (mean±SD) and paired t-test of n=3 repetitions Aβ (50 nM) indirect ELISA for di-scFv3D6-8D3 and [125I]di-scFv3D6-8D3 c mTfR1 competition ELISA of di-scFv3D6-8D3 and [125I]di-scFv3D6-8D3 d IC50 (mean ± SD) and paired t-test of n=3 repetitions mTfR1 competition ELISAs for di-scFv3D6-8D3 and [125I]di-scFv3D6-8D3. Fig. S4 125I Standards mean intensity ± SD of the plates used in the brain autoradiography experiments (1000 Bq n=7; 333 Bq n=7; 111 Bq n = 11). Fig. S5 Example overlay image. The accuracy of the thresholding in the NTE-image quantification was evaluated visually by applying the respective ROI outlines (NTE and CD31) as overlays with 50% opacity on the original composite image. Fig. S6 Color-inverted version of Fig. 7a-b, showing NTE (white puncta) detecting i.v. injected a [125I]mAb3D6-scFv8D3 or b [125I]di-scFv3D6-8D3 and CD31-flourescent staining (red) in mouse brain sections. [file 12987_2021_257_MOESM1_ESM.docx]

**Additional file 1**

**a b**


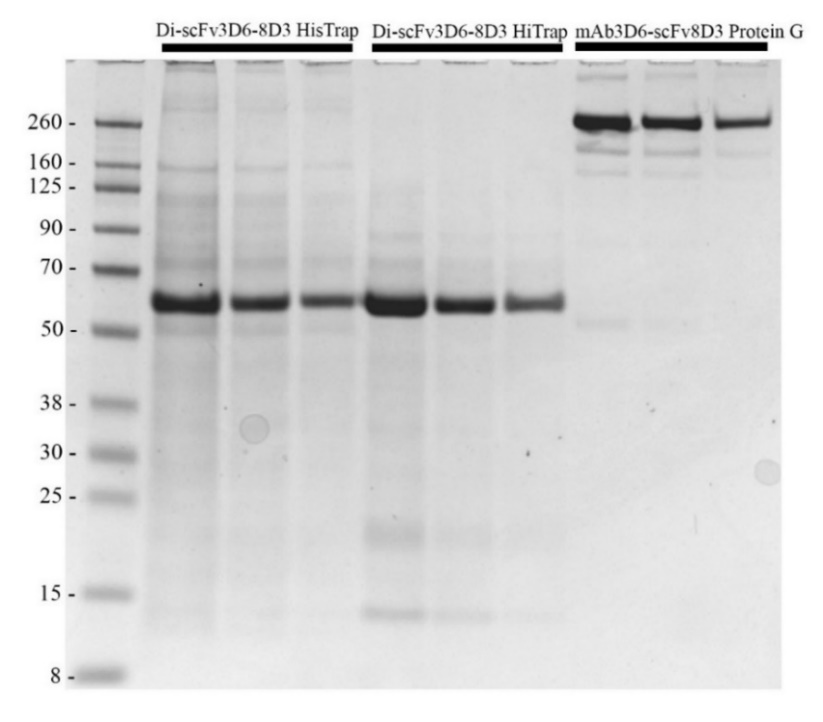

**Fig. S1 a** Non-reducing SDS-PAGE (4-12% Bis-Tris gel) of purified di-scFv3D6-8D3 and mAb3D6-scFv8D3. Lane 1 is protein ladder (Chameleon® Duo Pre-stained Protein ladder): lanes 2-4 is di-scFv3D6-8D3 purified by a HisTrap column; lanes 5-7 is di-scFv3D6-8D3 after HiTrap ion exchange chromatography; and lanes 8-10 is mAb3D6-scFv8D3 after purification by a Protein G column. For all proteins, 2, 1 and 0.5 μg was added in lanes 2-4, 5-7 and 8-10, respectively **b** Quantification of %Purity of lane 2 and 5, HMW = High molecular weight (>58 kDa) and LMW = low molecular weight (<58 kDa).

**ELISA**
All ELISA experiments were performed in half-area 96-well plates (Corning inc.). All coatings were done in phosphate buffer saline (PBS) overnight at 4°C. Coated plates were blocked with 1% BSA for 1 h at room temperature. All further antibody dilutions were made in ELISA incubation buffer (PBS with 0.1% BSA, 0.05% Tween, and 0.15% Proclin). Radiolabelled and non-radiolabelled mAb3D6-scFv8D3 and di-scFv3D6-8D3 were 5-fold serially diluted and incubated on plates overnight at 4°C. Bound antibody was detected with horseradish peroxidase (HRP)‑conjugated secondary antibodies; anti-mouse-IgG-F(ab’)_2_ (#115-035-006 Jackson ImmunoResearch Laboratories, West Grove, PA, USA) for mAb3D6-scFv8D3 and anti-His-Tag (#HRP66005, Proteintech Goup INC., IL, USA) for di-scFv3D6-8D3. K blue aqueous TMB substrate (Neogen Corp., Lexington, USA) was used for developing and 1M H_2_SO_4_ to stop the reaction. All plates were immediately read with a spectrophotometer at 450 nm.

The concentration of [^125^I]mAb3D6-scFv8D3 was determined by a sandwich ELISA, where plates were coated with 0.5 µg/ml anti-mouse-IgG (Vector Laboratories Inc, Burlinggame, CA). The effect of radiolabelling on Aβ-binding of mAb3D6-scFv8D3 and di-scFv3D6-8D3 was assessed using indirect ELISA on plates coated with 50 nM of Aβ_42_ (Innovagen, Lund, Sweden) (Fig. S2 a and Fig. S3 a). The effect of radiolabelling on the ability of mAb3D6-scFv8D3 to bind mTfR1 (BioArctic AB, Stockholm, Sweden) was assessed by indirect ELISA on plates coated with 13.3 nM mTfR1. A competition ELISA was used for [^125^I]di-scFv3D6-8D3, where plates were coated with 6.7 nM of mTfR1. Serially diluted non-labelled di-scFv3D6-8D3 and [^125^I]di-scFv3D6-8D3 were incubated with a constant 5 nM biotinylated di-scFv3D6-8D3. Then HRP-conjugated streptavidin (1:2000; Mabtech AB, Nacka, Sweden) was used for detection.


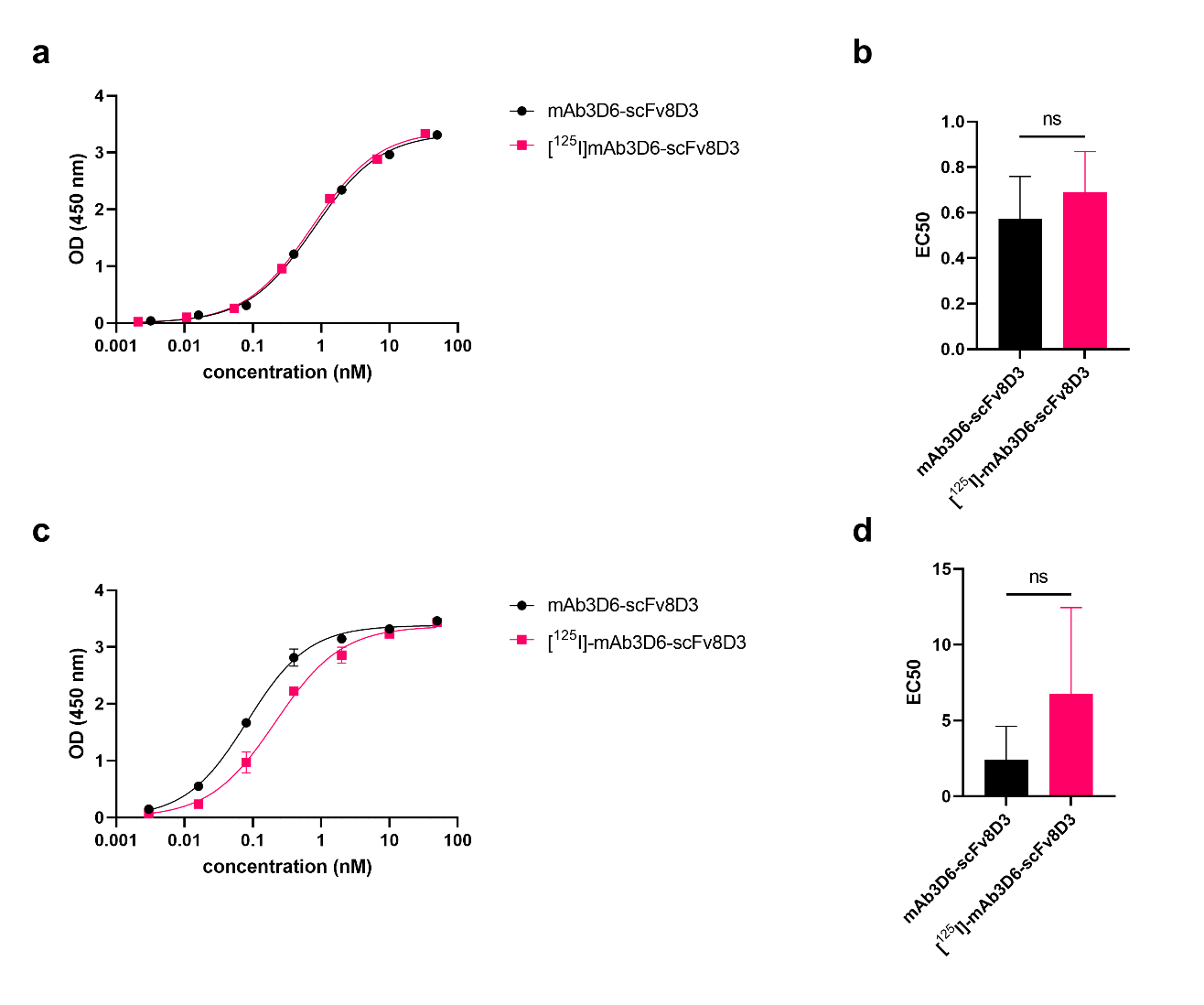

**Fig. S2** **a** Aβ_42_ (50 nM) indirect ELISA of mAb3D6-scFv8D3 and [^125^I]mAb3D6-scFv8D3 **b** EC50 (mean±SD) and paired t-test of n=3 repetitions Aβ (50 nM) indirect ELISA of mAb3D6-scFv8D3 and [^125^I]mAb3D6-scFv8D3 **c** mTfR1 (13.3 nM) indirect ELISA of mAb3D6-scFv8D3 and [^125^I]mAb3D6-scFv8D3 **c** EC50 (mean±SD) and paired t-test of n=3 repetitions mTfR1 (13.3 nM) indirect ELISA of mAb3D6-scFv8D3 and [^125^I]mAb3D6-scFv8D3

**Fig. S3** **a** (50 nM) indirect ELISA of di-scFv3D6-8D3 and [^125^I]di-scFv3D6-8D3 **b** EC50 **(**mean±SD) and paired t-test of n=3 repetitions Aβ (50 nM) indirect ELISA for di-scFv3D6-8D3 and [^125^I]di-scFv3D6-8D3 **c** mTfR1 competition ELISA of di-scFv3D6-8D3 and [^125^I]di-scFv3D6-8D3 **d** IC50 (mean ± SD) and paired t-test of n=3 repetitions mTfR1 competition ELISAs for di-scFv3D6-8D3 and [^125^I]di-scFv3D6-8D3.


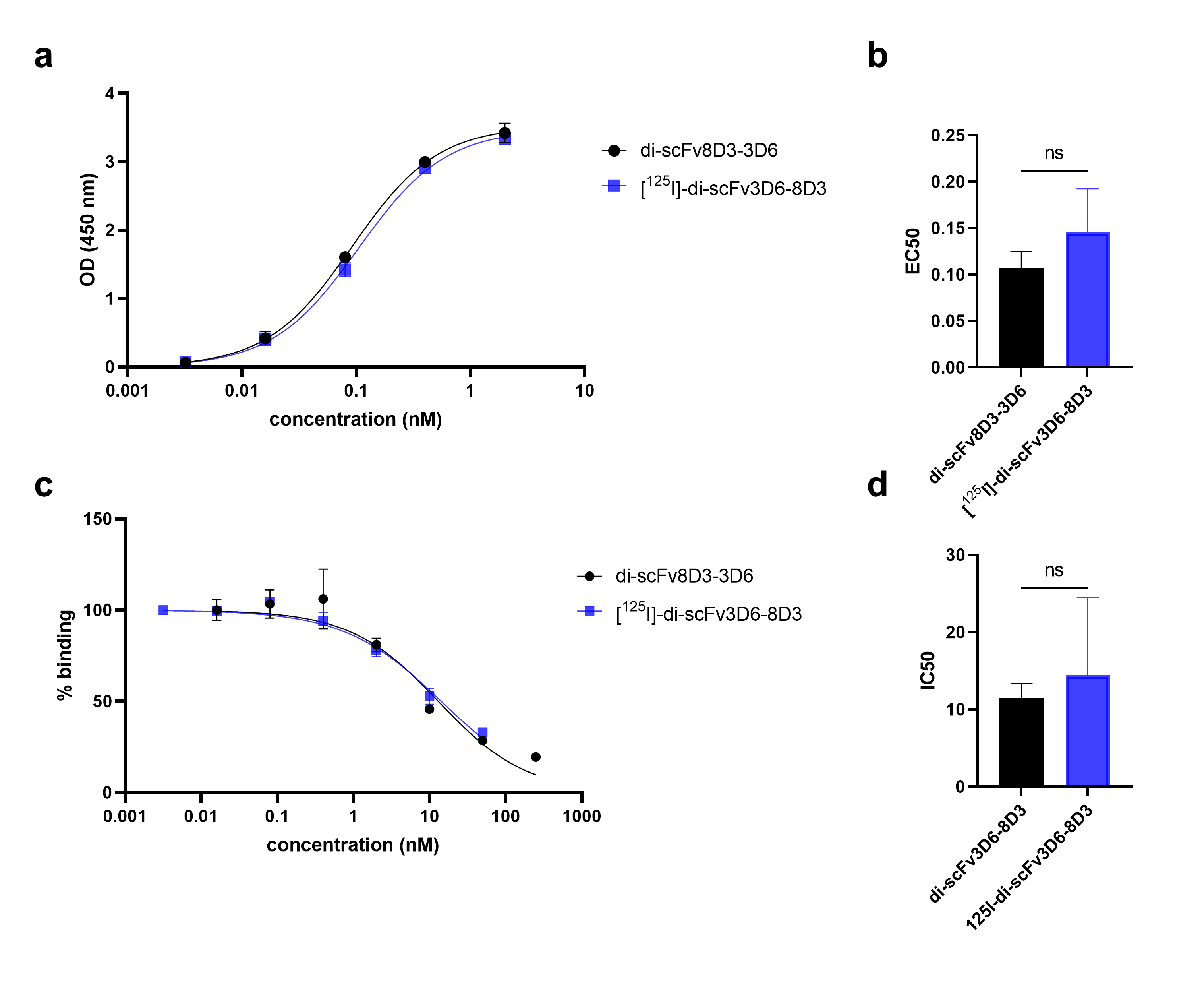


**Fig. S4** ^125^I Standards mean intensity ± SD of the plates used in the brain autoradiography experiments (1000 Bq n=7; 333 Bq n=7; 111 Bq n = 11).


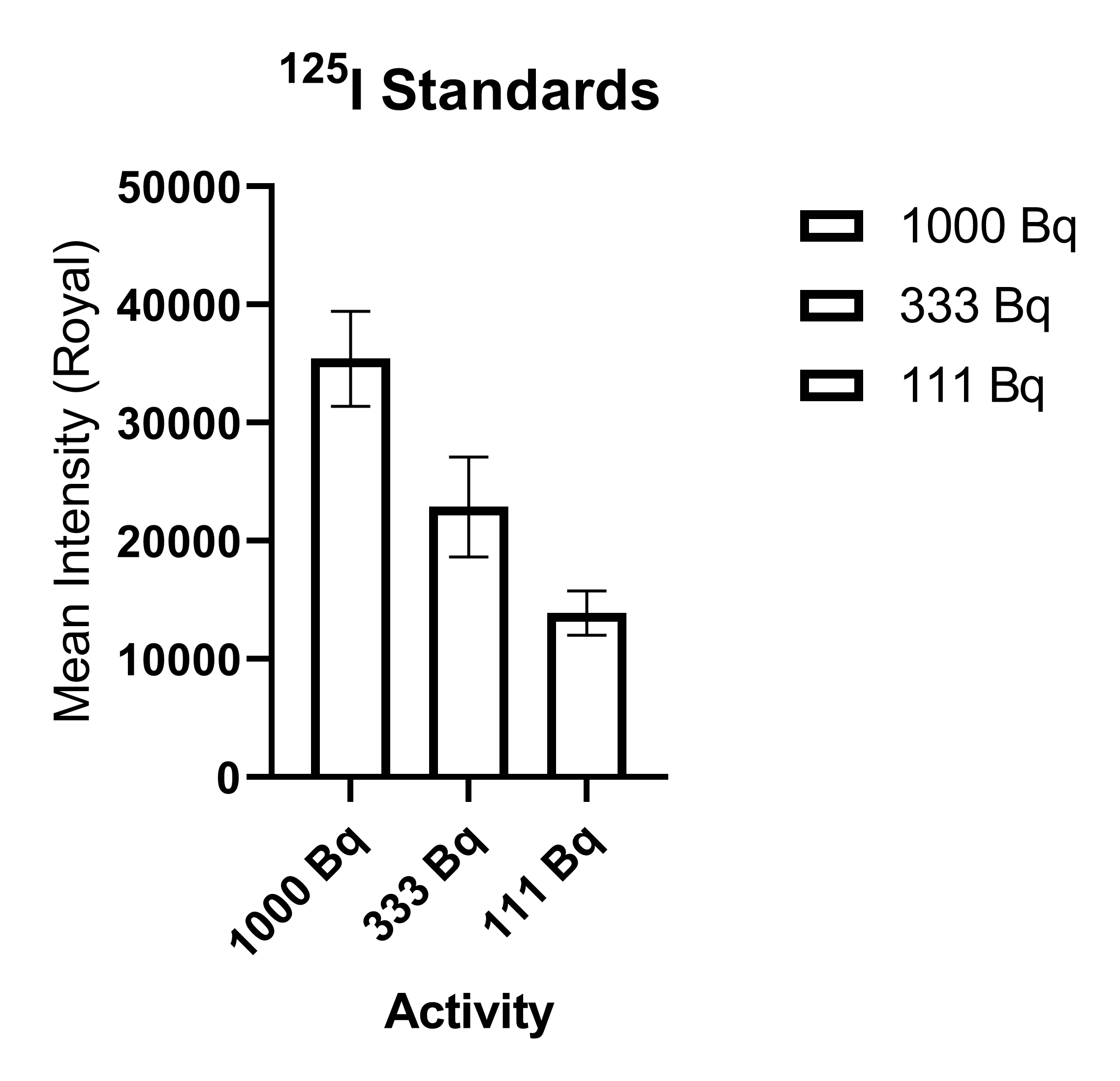


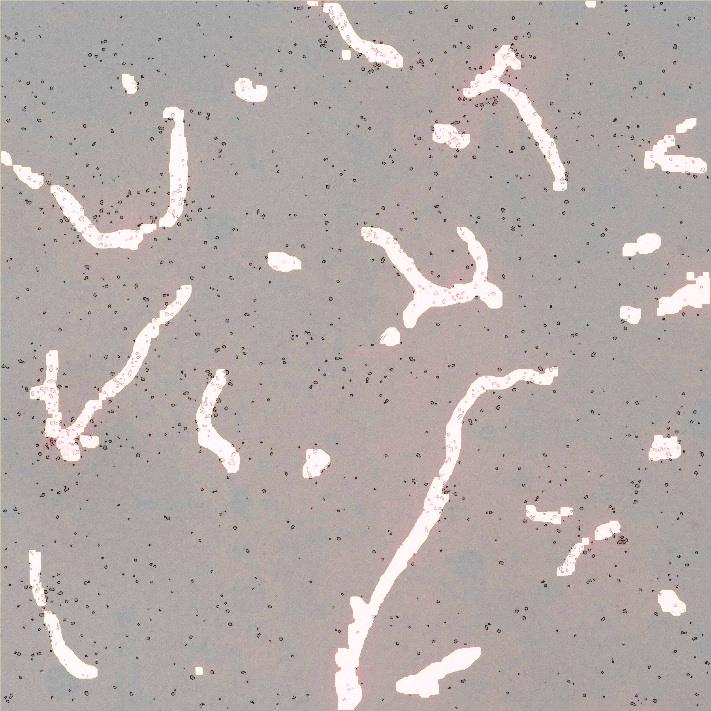

**Fig. S5** Example overlay image. The accuracy of the thresholding in the NTE-image quantification was evaluated visually by applying the respective ROI outlines (NTE and CD31) as overlays with 50% opacity on the original composite image.


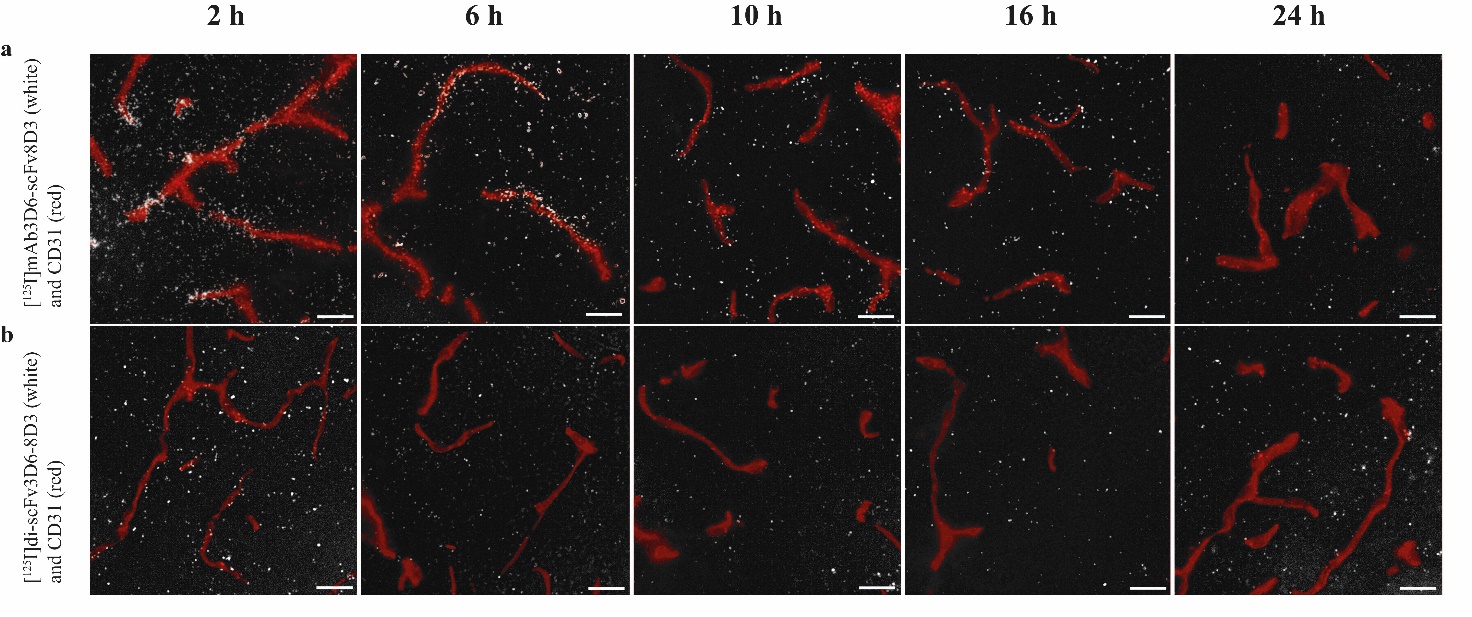


**Fig. S6** Color-inverted version of Figure 7 a-b, showing NTE (white puncta) detecting *i.v.* injected **a** [^125^I]mAb3D6-scFv8D3 or **b** [^125^I]di-scFv3D6-8D3 and CD31-flourescent staining (red) in mouse brain sections.
